# Case Report: Novel Heterozygous *DFNA5* Splicing Variant Responsible for Autosomal Dominant Non-syndromic Hearing Loss in a Chinese Family

**DOI:** 10.3389/fgene.2020.569284

**Published:** 2020-08-31

**Authors:** Xi Chen, Bao-Long Jia, Mei-Hui Li, Yuan Lyu, Cai-Xia Liu

**Affiliations:** Key Laboratory of Maternal-Fetal Medicine of Liaoning Province, Key Laboratory of Obstetrics and Gynecology of Higher Education of Liaoning Province, Department of Gynecology and Obstetrics, Liaoning Centre for Prenatal Diagnosis, Research Center of China Medical University Birth Cohort, Shengjing Hospital of China Medical University, Shenyang, China

**Keywords:** DFNA5, hearing loss, mutation, exome sequencing, ADNSHL

## Abstract

Autosomal dominant non-syndromic hearing loss (ADNSHL) has a broad phenotypic spectrum which includes bilateral, symmetrical, and high-frequency sensorineural hearing loss, that eventually progresses into hearing loss at all frequencies. Several genetic variations have been identified as causal factors underlying deafness, autosomal dominant 5 (*DFNA5*) gene-related hearing loss. Here, we report a novel mutation (c.991-1G > C) in *DFNA5*, which co-segregated with late-onset ADNSHL in a Chinese family and was identified via exome sequencing and Sanger sequencing of DNA from peripheral blood of the family members. Further sequencing of cDNA derived from peripheral blood mRNA revealed that the c.991-1G >C mutation led to the skipping of exon 8, which is a known pathogenic mechanism for DFNA5-related hearing loss.

## Introduction

Genetic hearing loss is a common congenital sensory disorder worldwide. In approximately 50% of cases, hearing loss is caused by genetic factors, and 70% of people with hereditary hearing loss are classified as cases of non-syndromic hearing loss, which is not associated with other diseases (Cunningham and Tucci, 2017). Autosomal dominant non-syndromic hearing loss (ADNSHL) accounts for approximately 20% of all cases of non-syndromic hereditary hearing loss and is generally characterized by a post-lingual onset and progressive hearing loss (Shearer et al., 1993). The phenotypic spectrum of ADNSHL includes bilateral, symmetrical, and high-frequency sensorineural hearing loss, which progresses further into hearing loss at all frequencies. Deafness, autosomal dominant 5 (*DFNA5*), which is known as *GSDME* in the context of cancer, is the fifth *DFNA* locus associated with ADNSHL. Numerous *DFNA5*-associated splice-site variations have been reported as pathogenic mutations for hearing loss. All these variations have a common effect of skipping exon 8 at the mRNA level, which results in an identical gain-of-function effect at the protein level. Here, we report a novel pathogenic *DFNA5* splice-site variant, which was identified in a Chinese family via exome sequencing and validated using Sanger sequencing. Our findings further support the pathogenicity of variants affecting the splicing of *DFNA5* exon 8.

## Case Description

### Pedigree and Clinical Evaluations

**FIGURE 1:**
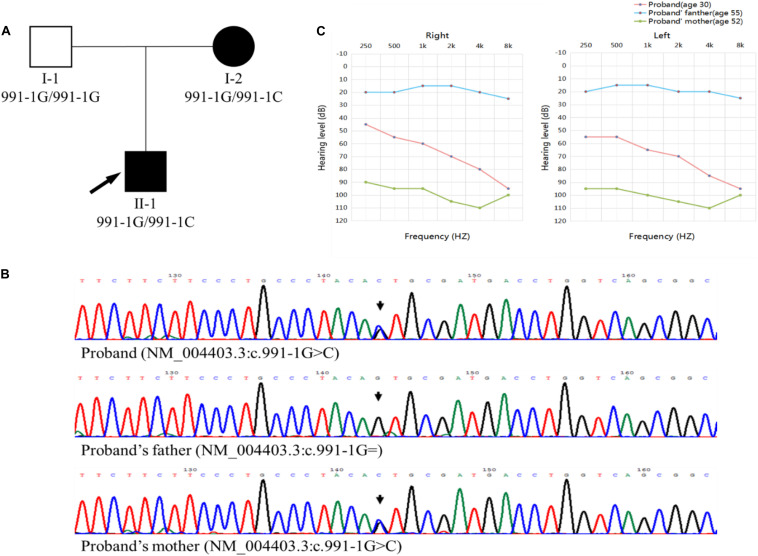
**(A)** Pedigree chart. The affected members are indicated by filled symbols, and the unaffected member is indicated by an open symbol. The arrow and indicates the proband. **(B)** Sanger sequencing chromatograms, identifying the c.991-1G >C mutation in the proband (II-1) and his mother (I-2) and wild-type c.991-1G in the proband’s father (I-1). **(C)** Audiograms of the family members. The charts present the data for the right and left ears of every participant, respectively. The red line represents the proband, and the blue and green lines represent proband’s father and mother, respectively.

We recruited three members of a Chinese family affected by late-onset ADNSHL, including the proband (II-1) and his parents (Figure 1A). Thus, the pedigree comprised two affected (proband and his mother) and one unaffected (proband’s father) family members. The pattern of inheritance was suggestive of ADNSHL. Based on the medical histories, no clear exposures could be suspected to have caused this hearing impairment. Therefore, the possibility of environmental causes or syndromic hearing impairment was excluded. The age of onset of hearing loss was recorded from the second to fourth decade because the proband’s mother (I-2) only had a vague memory of it.

All the family members agreed to undergo clinical evaluations, including a complete medical history and detailed physical examination. Auditory evaluations included otoscopic examination, otoacoustic emission, and pure-tone audiometry (PTA). All three members were subjected to air-conduction PTA, in which the hearing thresholds were determined at six frequencies (0.25, 0.5, 1, 2, 4, and 8 kHz). The same frequencies were used for bone-conduction PTA in the proband (II-1) and his mother (I-2). The severity of the impairment was judged as mild (20–40 dB HL), moderate (41–70 dB HL), severe (71–95 dB HL), and profound (>95 dB HL) hearing loss.

In the proband, the ADNSHL was bilateral and symmetric, varying from moderate to profound, whereas in the proband’s mother (I-2), the ADNSHL was profound (Figure 1C).

### Identification and Functional Characterization of the Splice-Site Variant

Peripheral blood samples were obtained from all the individuals with ethical approval. Genomic DNA was extracted from the blood samples using the TIANamp blood DNA kit (TIANGEN). The genomic DNA of the proband was subjected to exome sequencing. Targeted exon sequences and their flanking sequences were captured and enriched using an array-based hybridization chip (xGen Exome Research Panel v1.0; Integrated DNA Technologies, United States), followed by HiSeq X10 (Illumina) sequencing. All autosomal and sex chromosome variants were annotated using the ANNOVAR tool. The pathogenicity of the variants was annotated using the Human Gene Mutation Database^1^, ClinVar database^2^, and standard variants of the American College of Medical Genetics and Genomics (ACMG) (Richards et al., 2015). A series of *in silico* impact score procedures, including M-CAP^3^, SIFT^4^, Polyphen2^5^, LRT^6^, FATHMM^7^, and PROVEAN^8^, were used to prioritize all the variants according to ACMG guideline PP3. The variants were filtered using the Phenolyzer procedure^9^, with “deafness,” “hearing loss,” and “hearing impairment” used as the keywords. Variants with minor allele frequencies, <0.01, in any of the several databases queried (abSNP, ExAC, 1000 Genomes Project, gnomAD, and an in-house database) were selected for the analysis.

A *DFNA5* (GenBank: AF073308) gene fragment was amplified and sequenced using a pair of human *DFNA5*-specific primers (forward 5′-TGTAGCCACCAAGGATTAGCAA-3′ and reverse 5′-AGGGCACTGACCAAGAAGTAGG-3′). Sanger sequencing for all family members was conducted on the ABI 3730 platform (Applied Biosystems) and confirmed the presence of the c.991-1G >C mutation in *DFNA5* (NM_004403.3), which co-segregated with the disease phenotype (Figure 1B). Exome sequencing did not reveal any other possible disease-causing variations or modifier genes.

**FIGURE 2:**
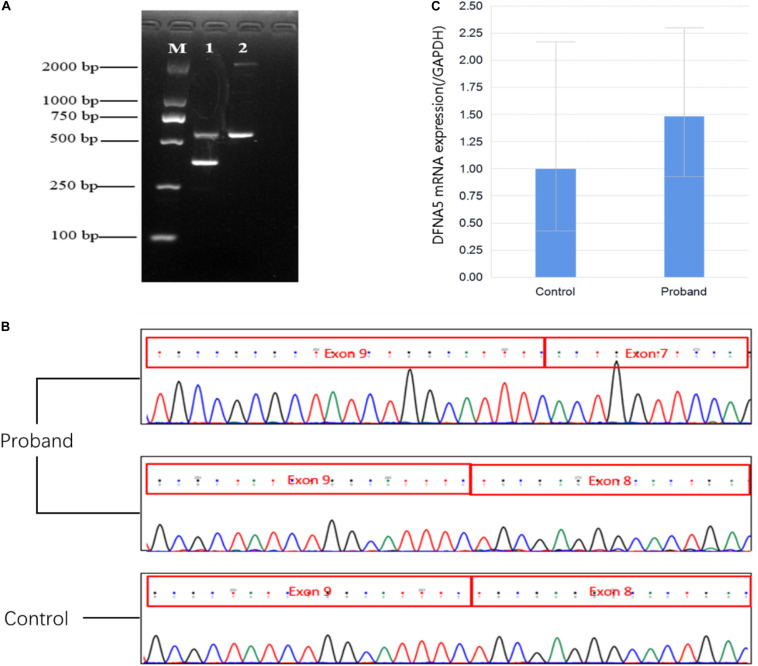
**(A)** Agarose gel electrophoresis. M: 2,000-bp marker; 1: cDNA product of the proband; 2: cDNA product of the control. **(B)** Sanger sequencing chromatograms of the cDNA products. The proband sample shows a sequence of the 392-bp fragment, indicating the connection of exon 9 and exon 7 and a sequence of the 585-bp fragment, indicating the connection of exon 9 and exon 8; the control sample shows a sequence of the 585-bp fragment, indicating the connection of exon 9 and exon 8. **(C)** Quantification of the qPCR products of *DFNA5* mRNA. The *DFNA5* mRNA expression level in the proband was 1.482-fold of that in the control.

To investigate the potential effect of the c.991-1G >C mutation in *DFNA5* on mRNA splicing at exon 8, primers were designed for the sequences of exon 7 (reverse 5′-TTTCCATCCATTTGCGGAGC-3′) and exon 10 (forward 5′- GCACAGAGTCCATTCAGGGT-3′) and used to amplify a fragment of cDNA generated from peripheral blood mRNA. mRNA was extracted using the RNAprep Pure tissue kit (TIANGEN), and was reverse transcribed to cDNA using the RevertAid first-strand cDNA synthesis kit (Thermo Fisher Scientific, Waltham, MA, United States). Polymerase chain reaction (PCR) was conducted using the Phanta Max super-fidelity DNA polymerase (Vazyme Biotech). The amplified fragments were sequenced on the ABI 3730 platform. Agarose gel electrophoresis of the PCR products revealed fragments of 585 and 392 bp in the proband cDNA and a 585-bp fragment in the control (Figure 2A). Sequence analysis of the aberrant PCR product confirmed the skipping of exon 8 in the mutant transcript, resulting in a direct connection between exons 7 and 9 (Figure 2B). Skipping of exon 8 led to a translational frameshift and a premature termination codon (PTC) in exon 10. The PTC in the last exon of the abnormal transcript could not lead to nonsense-mediated mRNA decay (Kim et al., 2013; White et al., 2015). qPCR was conducted using the KAPA SYBR FAST universal 2 × qPCR master mix (KAPA, MA, United States), with primers designed against the *DFNA5* coding sequence and located upstream of the variant: forward 5′-CGCCTGGAAGATGTCACTCA-3′ (exon 7) and reverse 5′-ACTCTGTCTACCTGGACCCC-3′ (exon 6). The results of qPCR revealed that the relative expression of *DFNA5* mRNA in the proband was 1.482-fold of that in the control (Figure 2C).

## Discussion

All *DFNA5* mutations reported to date are splice-site variants leading to the skipping of exon 8 (Table 1; Van Laer et al., 1998; Yu et al., 2003; Bischoff et al., 2004; Cheng et al., 2007; Park et al., 2010; Chai et al., 2014; Nishio et al., 2014; Li-Yang et al., 2015; Nadol et al., 2015; Booth et al., 2018; Wang et al., 2018; Booth et al., 2020). The common phenotype in families with a *DFNA5* mutation is bilateral, symmetrical, late-onset hearing loss, starting at high frequencies and ultimately progressing to involve all frequencies, with the severity ranging from moderate to profound. Consistently, the affected family members evaluated in this study showed these typical clinical symptoms. In addition to the previously reported c.991-2A >G mutation, which is located at the conventional AG/GT splice site (Chai et al., 2014), we identified a mutation, c.991-1G >C, located in the same region, at the boundary of intron 7 and exon 8, which was confirmed to abolish the splicing of exon 8.

**TABLE 1:** Summary of reported *DFNA5* variations.

**cDNA mutation**	**Location**	**Mutation effect**	**Phenotype of the hearing impairment**	**Country/Region**	**References**
c.990+503_990+1691delins132	Intron 7	Skipping of exon 8	High–all frequencies	Netherlands	Van Laer et al., 1998
c.991–6C > G	Intron 7	Skipping of exon 8	High–all frequencies	Netherlands	Bischoff et al., 2004
c.991–3C > A	Intron 7	Skipping of exon 8	High–all frequencies	China	Wang et al., 2018
c.991–2A > G	Intron 7	Skipping of exon 8	High frequencies	China	Chai et al., 2014
	Intron 7	Skipping of exon 8	High–all frequencies	Europe	Booth et al., 2018
c.991–15_991–13del	Intron 7	Skipping of exon 8	High frequencies	China	Yu et al., 2003
	Intron 7	Skipping of exon 8	High frequencies	Korea	Park et al., 2010
	Intron 7	Skipping of exon 8	High frequencies	Japan	Nishio et al., 2014
	Intron 7	Skipping of exon 8	High frequencies	United States	Nadol et al., 2015
	Intron 7	Skipping of exon 8	All frequencies	China	Wang et al., 2018
	Intron 7	Skipping of exon 8	High frequencies	East Asia	Booth et al., 2018
	Intron 7	Skipping of exon 8	High–all frequencies	Europe	Booth et al., 2020
c.1183G > A	Exon 8	Skipping of exon 8	High frequencies	East Asia	Booth et al., 2018
c.1183+4A > G	Intron 8	Skipping of exon 8	High frequencies	China	Cheng et al., 2007
c.1183+1delG	Intron 8	Skipping of exon 8	High–all frequencies	China	Li-Yang et al., 2015
c.1154C > T	Exon 8	Skipping of exon 8	High frequencies	Iran	Booth et al., 2018
c.1102C > G	Exon 8	Skipping of exon 8	High frequencies	Europe	Booth et al., 2018
c.991–1G > C	Intron 7	Skipping of exon 8	High–all frequencies	China	Present study

*DFNA5* encodes a protein of 496 amino acids, which is expressed in the human cochlea (Van Laer et al., 1998). Skipping of exon 8 at the mRNA level leads to premature termination of the encoded protein, which results in cochlear hair cell loss via apoptosis (Rogers et al., 2017). The DFNA5 protein has an N-terminal domain, with apoptosis-inducing activity, and a C-terminal domain, which folds back to shield the N-terminus and prevent inappropriate initiation of apoptosis (Li et al., 2019). Skipping of exon 8 in *DFNA5* transcripts results in the translation of a truncated protein with apoptosis-inducing activity, owing to the loss of the protective C-terminal domain. *In vitro*, a cDNA construct with deleted exon 8 was shown to induce programmed cell death in both human and yeast cell lines (Van Rossom et al., 2015). However, *Dfna5^–/–^* and *Dfna5^+/–^* knockout mice did not exhibit hearing loss, despite a divergent number of cochlear fourth-row outer hair cells (Van Laer et al., 2005). The principal histopathological features of human ADNSHL include the loss of inner and outer hair cells, along with severe degeneration of the stria vascularis and spiral ligament throughout the cochlea (Nadol et al., 2015).

In conclusion, we identified a novel *DFNA5* splicing variant, c.991-1G >C, in a Chinese family. This finding supports the pathogenicity of variants that affect the splicing of *DFNA5* exon 8.

## Data Availability Statement

The datasets for this article are not publicly available due to concerns regarding participant/patient anonymity. Requests to access the datasets should be directed to the C-XL, liucx1716@163.com.

## Ethics Statement

This study was approved by the Medicine Ethics Committee of the Shengjing Hospital of China Medical University. The patients/participants provided their written informed consent to participate in this study. Written informed consent was obtained from the proband and his parents for the publication of any potentially identifiable images or data included in this article.

## Author Contributions

XC and YL conceived and designed the experiments. M-HL, YL, and C-XL helped with the patient’s workup and recruitment of the patient and his family members. XC, B-LJ, and M-HL performed the experiments and helped with genetic analysis. XC, B-LJ, and YL wrote the manuscript. All authors contributed to the article and approved the submitted version.

## Conflict of Interest

The authors declare that the research was conducted in the absence of any commercial or financial relationships that could be construed as a potential conflict of interest.

## Footnotes

1 http://www.hgmd.cf.ac.uk/ac/index.php

2 http://www.ncbi.nlm.gov/clinvar/

3 http://bejerano.stanford.edu/MCAP/

4 http://sift.bii.a-star.edu.sg/

5 http://genetics.bwh.harvard.edu/pph2/

6 http://cadd.gs.washington.edu/

7 http://fathmm.biocompute.org.uk/inherited.html

8 http://provean.jcvi.org/index.php

9 http://phenolyzer.wglab.org/
